# Surface Acoustic Wave (SAW) Vibration Sensors

**DOI:** 10.3390/s111211809

**Published:** 2011-12-19

**Authors:** Jerzy Filipiak, Lech Solarz, Grzegorz Steczko

**Affiliations:** 1 Institute of Electronic and Control Systems, Technical University of Czestochowa, 17 A.K. Str., 42-200 Częstochowa, Poland; E-Mails: filipiak@el.pcz.czest.pl (J.F.); gem@gemsc.com.pl (G.S.); 2 Department of Civil Engineering, Military University of Technology, 2 Kaliskiego Str., 00-908 Warsaw, Poland

**Keywords:** surface acoustic waves, vibration sensor, delay line, mechanical vibration, electronic warning system

## Abstract

In the paper a feasibility study on the use of surface acoustic wave (SAW) vibration sensors for electronic warning systems is presented. The system is assembled from concatenated SAW vibration sensors based on a SAW delay line manufactured on a surface of a piezoelectric plate. Vibrations of the plate are transformed into electric signals that allow identification of the sensor and localization of a threat. The theoretical study of sensor vibrations leads us to the simple isotropic model with one degree of freedom. This model allowed an explicit description of the sensor plate movement and identification of the vibrating sensor. Analysis of frequency response of the ST-cut quartz sensor plate and a damping speed of its impulse response has been conducted. The analysis above was the basis to determine the ranges of parameters for vibrating plates to be useful in electronic warning systems. Generally, operation of electronic warning systems with SAW vibration sensors is based on the analysis of signal phase changes at the working frequency of delay line after being transmitted via two circuits of concatenated four-terminal networks. Frequencies of phase changes are equal to resonance frequencies of vibrating plates of sensors. The amplitude of these phase changes is proportional to the amplitude of vibrations of a sensor plate. Both pieces of information may be sent and recorded jointly by a simple electrical unit.

## 1. Introduction

One of the possible applications of surface acoustic waves (SAWs) elements are sensors for different physical quantities. SAW sensors can be used to measure gas concentration [1–4], temperature, pressure [5–9] and mechanical quantities: moment (of force) in shaft rotation [10], tension [11–13], acceleration [14,15] and vibrations [16].

The principle of operation of all SAW sensors is a measurement of an alteration in surface wave delay caused by the influence of measured physical quantity on the speed and propagation path length of a wave. That is why the construction of all such devices requires dealing with similar issues. However, some important issues characteristic of a group of sensors designed to measure some particular physical quantity may arise, depending on the type of quantity to be measured. For example, to create sensors designed to measure mechanical quantities it is necessary to solve an exemplary mechanical unit of a sensor. Moreover, it is also essential to prepare a simulation of an electrical-to-mechanical transducer and a simulation of an electrical unit cooperating with the sensor. The first issue mentioned is only relevant to SAW vibration and acceleration sensors.

**Figure 1.**
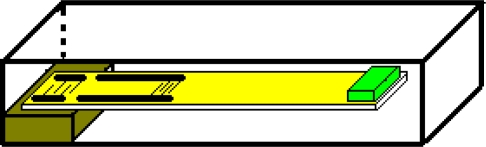
Basic structure of a SAW vibration sensor.

In this work a SAW vibration sensor, designed specifically to be used in an electronic warning system is presented. The basic structure of a SAW vibration sensor is shown in Figure 1. The main part is an anisotropic plate made of a piezoelectric material. One side of the plate is stiffly attached to the sensor casing, while the other free side is loaded with the seismic mass. On the upper surface of the plate a SAW delay line in the form of a four-terminal network has been made.

Any motion of the casing of a sensor creates vibrations of a plate and an alteration in the time delay of a SAW delay line. It results in a high frequency signal phase which goes through such a line. The magnitude of an alteration of signal phase is proportional to the alteration in time delay of the SAW delay line. The frequency of the phase change is equal to the vibration frequency of the sensor plate.

The sensor plate is a continuous mechanical unit. Such a unit may, theoretically, possess an infinite number of natural vibration frequencies. A vibration sensor designed for an electronic warning system should have only one frequency of free vibration [14]. This allows the identification of a vibrating sensor.

The amplitude of high frequency signal phase changes will be proportional to the amplitude of vibrations of a sensor plate. Its magnitude may be used to determine the state of motion of a base to which sensor casing is fixed. It raises a basic question: is it possible to create a sensor shown in Figure 1 that will fulfill all of these requirements? The answer may be obtained as a result of an analysis of the dynamics of a movement of a sensor plate. The preparation of the analysis requires a mechanical model of a sensor. The mechanical properties of sensor plate determine the technical parameters of the mechanical sensors. The strength of a material determines the operating range of every sensor. In the case of movement of a sensor plate its mechanical properties are described by its frequency response (amplitude and phase). They determine the sensitivity, linearity, delay, operating range and accuracy of a sensor. That is why the next section will be devoted to a model of a mechanical unit of a sensor. The SAW vibration sensor is compared with micromechanical acceleration sensors based on silicon (Micro Electro Mechanical Systems accelerometers) and piezoelectric accelerometers in the Conclusions section.

## 2. Model of Mechanical Unit for SAW Vibration Sensor

**Figure 2.**
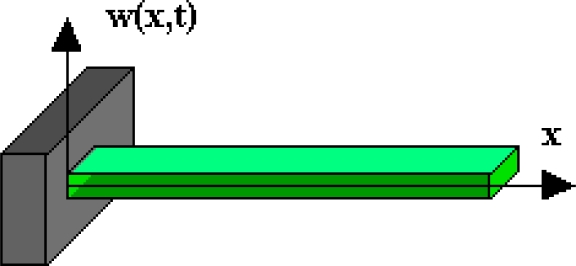
The plate of a vibration sensor.

The object of consideration has been presented in Figure 2. One end of the plate is stiffly attached, and the other is free and without any concentrated mass. The piezoelectric properties of sensor plate will be omitted in the analysis.

The equation of a movement of an anisotropic body with the mass density *ρ* is:

(1) $$\frac{{\partial \sigma }_{\mathrm{ij}}}{{\partial x}_{j}}+\rho \frac{\partial^{2}u_{i}}{{\partial t}^{2}}=0$$

The stress tensor *σ_ij_* depends on the strain tensor *ε_kl_* through Hook-Voight equation:

(2a) $$\sigma_{\mathrm{ij}}=C_{\mathrm{ijkl}}\cdot \varepsilon_{\mathrm{kl}}+\Sigma_{\mathrm{ijkl}}\cdot \frac{{\partial \varepsilon }_{\mathrm{kl}}}{\partial t}$$

where: *C_ijkl_*—is a elasticity tensor, Σ*_ijkl_*—is a material damping tensor, *u_i_*—is a displacement vector,

(2b) $$\varepsilon_{\mathrm{kl}}=\frac{1}{2}\left(\frac{{\partial u}_{k}}{{\partial x}_{l}}+\frac{{\partial u}_{l}}{{\partial x}_{k}}\right)\;-\text{is a strain tensor}$$

The mathematical description of this issue will be closed if the initial and boundary conditions are added to the aforementioned equations. Solving this problem is complicated. The causes of the complications are the huge number of non-vanishing modules of an elasticity and material damping tensor. The material damping tensor *Σ_ijkl_* has an identical symmetry as the elasticity tensor *C_ijkl_*. For materials typically used in SAW devices (quartz, lithium niobate), the value of damping constants are difficult to verify experimentally. For the higher class of symmetry of an anisotropic material, Equations (1) and (2) are simpler. For the isotropic material the elasticity tensor has only two independent components. therefore the elastic properties of an isotropic substance describe two quantities. They are often shown in form of a Young’s modulus *E* and a Poisson ratio *ν*. The following relations occur between quantities *E*, *ν*, and elasticity tensor components [17]:

(3) $$E=C_{1111},\;\;\;\;\;\;\;\;\;\nu \;=\frac{-C_{1122}}{C_{1111}+C_{1122}}$$

The description of viscous properties of an isotropic body done by material damping tensor is analogical. It is usually described by two quantities [17]:

(4) $$\tau =\frac{\Sigma_{1111}}{E},\;\;\;\;\;\;\eta =\frac{-\Sigma_{1122}}{\Sigma_{1111}+\Sigma_{1122}}$$

The Young's modulus *E* is described as a proportion of a longitudinal stress to longitudinal strain for the direction of the functioning of a stress. To describe the mechanical properties of anisotropic materials taking into account it calla of symmetry and a particular direction of the stress a effective Young's modulus may be used *E* [17,18]. An exemplary expression for a inverse effective Young's modulus for a trigonal unit (lithium niobate, quartz) is:

(5) $$1/E={\left(1-l_{3}^{2}\right)}^{2}s_{11}+l_{3}^{4}s_{33}+l_{3}^{2}(1-l_{3}^{2})(2s_{13}+s_{44})+2l_{2}^{}l_{3}^{}(3l_{1}^{2}-l_{2}^{2})s_{14}$$

where: *s_ij_*—is an element of an compliance matrix, *l_j_*—is a cosine of an angle between the chosen direction and the axis—*j*, in Cartesian coordinates.

The compliance matrix *s_ij_* is the reverse of the stiffness elasticity matrix *c_ij_*. It is possible to calculate the values of material damping coefficients in a chosen crystallographic direction, too. The presented approach allows one to model the anisotropic material using the isotropic model. In such a model the stresses are the sum of elastic and dissipative components:

(6) $$\sigma =E\varepsilon +E\tau \frac{\partial \varepsilon }{\partial t}$$

We consider equivalent isotropic model of cylindrically bent plate [19]. Equation of free vibrations has the form:

(7a) $$\rho \frac{\partial^{2}w(x,t)}{\partial t^{2}}+E_{e}\frac{h^{2}}{12}\left(1+\tau \frac{\partial }{\partial t}\right)\frac{\partial^{4}w(x,t)}{\partial x^{4}}=0$$

where *ρ*—mass density; *h*—plate thickness; *L*—plate length; *τ*—equivalent material damping coefficient,

(7b) $$E_{e}=\frac{E}{1-\nu^{2}}$$

*E_e_* is an equivalent Young’s modulus.

At the boundaries we have:

(8) $$\begin{array}{cccc} w(0,t)=0, & \frac{\partial w(0,t)}{\partial x}=0, & \frac{\partial^{2}w(L,t)}{\partial x^{2}}=0, & \frac{\partial^{3}w(L,t)}{\partial x^{3}}=0, \end{array}$$

The solution to the boundary problem Equations (7) and (8) has the form:

(9) $$w(x,t)=\sum_{n=1}^{\infty }W_{n}(x)\cdot A_{n}\cdot e^{-\frac{\varpi_{n}^{2}}{2}t}\cdot \;\text{sin}({\bar{\omega }}_{n}t+\varphi_{n})$$

where: constants *A_n_* and *φ_n_* are determined by initial conditions.

The angular frequency of non-damped vibrations is equal to:

(10) $$\omega_{n}=k_{n}^{2}\frac{h}{l^{2}}\sqrt{\frac{E_{e}}{12\rho }}$$

The angular frequency of damped vibrations is equal to:

(11) $${\bar{\omega }}_{m}=\;\omega_{m}\sqrt{1-\frac{\omega_{m}^{2}\tau^{2}}{4}}$$

where:

(12) $$k_{1}=1,875\;\;\;\;\;k_{2}=4,694\;\;\;\;\;k_{3}=7,855$$

The orthonormal set of function W_n_ (eigenfunctions) is taken from [19]. Only some elements in the sum Equation (9) represent vibrations. For *N* < *n*, where *N* is the greatest natural number for which *ω_N_* < 2/τ, the sum element represents very strongly damped movement and there is no resonance at this frequency. Each of the harmonics *n* = 1, 2, 3, ..... has part of the energy. How great is the part depends on the unit [normal vibrations, *ω_n_*, *w*(*x*)] and depends on activation.

**Figure 3.**
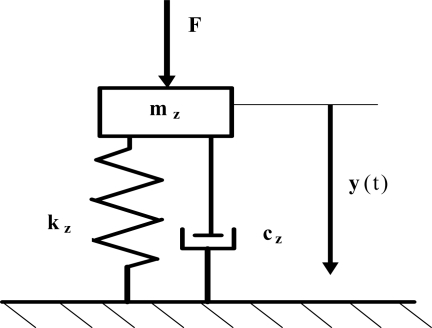
The equivalent circuit of sensor plate with one degree of freedom.

In [20] a simplified model with one degree of freedom was presented and it is shown in Figure 3. It has been used to describe the dynamics of sensor plate movement. It was derived according to the Rayleigh method. This method is based on a simplified modeling of a plate with the use of an equivalent circuit with one degree of freedom which is energetically equivalent. The free end of sensor plate has been taken as a point of reduction.

Parameters in the model are as follows [21]:

(13) $$m_{z}=0.25196\rho bhl,\;\;\;\;\;\;\;\;\;\;\;\;\;\;\;\;\;\;\;\;\;\;\;\;\;k_{z}=3.1169\frac{E_{e}\;b\;h^{3}}{l^{3}},\;\;\;\;\;\;\;\;\;\;\;\;\;\;\;\;\;\;\;\;\;\;\;\;\;c_{z}=\tau 3.1169\frac{E_{e}\;b\;h^{3}}{l^{3}}$$

The model with one degree of freedom has only one resonance frequency. The equation of mass movement is as follows:

(14) $$\frac{d^{2}y(t)}{{\mathrm{dt}}^{2}}+\omega_{0}^{2}\tau \frac{\mathrm{dy}(t)}{\mathrm{dt}}+\omega_{0}^{2}y(t)=\frac{F(t)}{m_{z}}$$

The solution of an equation for natural vibrations reads as follows:

(15) $$y(t)={\mathrm{Ae}}^{-\frac{\omega_{0}^{2}\tau }{2}t}\;\text{sin}(\omega_{r}t+\varphi )$$

where:

(16) $$\omega_{0}=3.5172\left(\frac{h}{l^{2}}\right)\;\sqrt{\frac{E_{e}}{12\;\rho }}$$

(17) $$\omega_{r}=\omega_{0}\sqrt{1-\frac{\omega_{0}^{2}\tau^{2}}{4}}$$

This is analogous to the Equation (10) obtained with the use of an isotropic model of a sensor plate. Comparison of the first frequency of the damped vibrations of the plate obtained in an isotropic model Equation (11) and the frequency of damped vibrations obtained with the use of a model with one degree of freedom Equation (17) fulfills the relation:

(18) $$\omega_{1}=0.9996\omega_{r}$$

The first frequency of damped vibrations calculated in an isotropic model is 0.5 per cent lower than frequency calculated with the use of a discrete model. This difference could be smaller in case of a sensor construction with the concentrated mass attached to the movable end of the plate. That is why the model with one degree of freedom may be used to describe the movement of a sensor plate. It allows relatively easy simulation of vibrations of the plate with the mass attached to its movable end. Free vibrations of sensor plate are defined as a sum of damped harmonic frequency vibrations, but in free vibration damped vibrations with first harmonic frequency will dominate. The amplitudes of the superior harmonic vibrations will be extremely small. As it is shown in [14] their quantity is 40 dB smaller than the first harmonic amplitude.

This is the reason why a model with one degree of freedom [14,20,21] has been used to analyze the movement of the plate with concentrated mass. Vibrations of the plate have been activated by the movement of the sensor casing *Y(t)*. The equation of movement is as following:

(19) $$\frac{d^{2}y(t)}{dt^{2}}+\omega_{0}^{2}\tau \frac{\mathrm{dy}(t)}{\mathrm{dt}}+\omega_{0}^{2}y(t)=\omega_{0}^{2}\tau \frac{\mathrm{dY}(t)}{\mathrm{dt}}+\omega_{0}^{2}Y(t)$$

where:

(20) $$\omega_{0}=3.5172\left(\frac{h}{l^{2}}\right)\;\sqrt{\frac{E_{e}}{12\rho }}\;\sqrt{\frac{1}{1+r\;3.9689}}$$

*r*—is a ratio between seismic mass and mass of sensor plate.

The solution of the Equation (19) is a function:

(21) $$\begin{array}{c} y(t)=A\;\text{exp}[-\frac{\omega_{0}^{2}\tau t}{2}]\;\text{sin}[\omega_{r}(t+\varphi )]-\\ -\frac{4\omega_{r}}{4-\omega_{0}^{2}\tau^{2}}\int_{0}^{t}\left(\tau \frac{\mathrm{dY}(\xi )}{\mathrm{dt}}+Y(\xi )\right)\cdot \text{exp}[-\frac{\omega_{0}^{2}\tau }{2}(t-\xi )]\cdot \text{sin}[\omega_{r}(1-\xi )]d\xi \end{array}$$

where: constants *A* and *φ* are determined by initial conditions.

Relations between ω_0_ and ω_r_ are as in identity [14]. In both components of the solution Equation (21) the following function appears:

(22) $$\phi_{\delta }(t)={\mathrm{Ae}}^{-\frac{\omega_{0}^{2}\tau }{2}t}\;\text{sin}(\omega_{r}t+\phi )$$

This is a product of a harmonic and damping (exponentially decay with time) function. The frequency of a harmonic function is the resonance frequency of the unit. This function describes sensor impulse response and its natural vibrations. It is a sum of:

- convolution of an impulse response of the plate and the component of describing movement of the sensor casing,
- damped vibrations with the resonance frequency of a sensor plate.

It will always have a factor in the form of a harmonic function with the frequency equal to the resonance frequency of sensor plate and with variable amplitude. That is why the frequency response of sensor plate may be quantity identifying the sensor. The frequency response of the sensor plate is the ratio of the amplitude of the deflection plate sensor to the harmonic amplitude of its case The frequency response of the sensor plate calculated from the Equation (19) is as follows:

(23) $$H(\omega )=\sqrt{\frac{1+{\left(\omega \tau \right)}^{2}}{{\left[1-{\left(\frac{\omega }{\omega_{r}}\right)}^{2}\right]}^{2}+{\left(\omega \tau \right)}^{2}}}$$

Parameters in relation Equation (23) depends on mechanical properties of the sensor plate material. The quantities of elastic and viscous parameters for quartz are shown in Table 1 [22,23].

**Figure 4.**
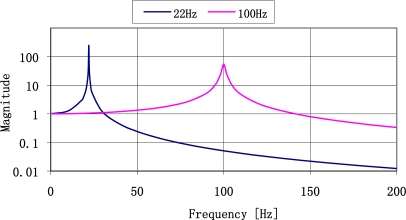
Theoretical resonance characteristics of plates with resonance frequencies of 22 Hz and 100 Hz.

Theoretical frequency response for plates made of ST-cut quartz with the resonance frequencies of 22 Hz and 100 Hz are shown in Figure 4. The most important is that for low frequency the frequency response has narrower band and higher magnitude so the selectivity of the sensor is high. It decreases with increased resonance frequency.

The maximum value of the frequency response of the plate will occur for *ω* = *ω_r_*. It has been described with the relationship:

(24) $$H(\omega_{r})=\sqrt{\frac{1+{\left(\omega_{r}\tau \right)}^{2}}{{\left(0,5\omega_{0}\tau \right)}^{4}+{\left(\omega_{r}\tau \right)}^{2}}}$$

**Figure 5.**
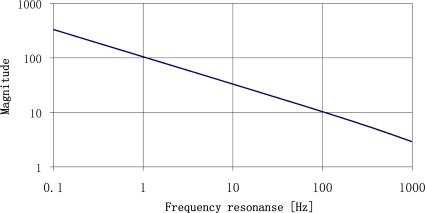
Maximum magnitude of frequency response *versus* the resonance frequency of the sensor plate.

Its value exceeds repeatedly the value of static deflection (e.g., for the resonance frequency of 22 Hz it is 246 times higher). The change in maximum magnitude of frequency response as a function of resonance frequency of the plate is shown in Figure 5.

For the resonance frequency of a plate of 10 Hz the vibrations amplitude multiplication is 1,600 higher than the static deflection. This property may be used to construct sensors with high sensitivity levels, but it is necessary to answer one question beforehand: what is the lowest possible resonance frequency of a plate that we can manufacture? The answer is accessible on the basis of the described model and the length of available plates. The resonance frequency of sensor plate is described by Equation (20). It depends on plate length (l) and on quantity of a concentrated mass (r) attached to the free end of sensor plate.

**Figure 6.**
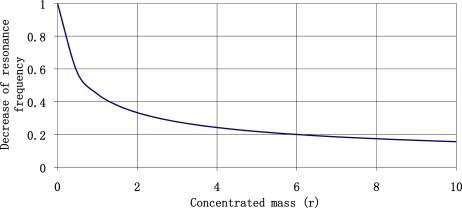
The influence of a change of a concentrated mass on sensor plate resonance frequency.

The increase of the concentrated mass lowers resonance frequency of the plate, simultaneously increasing stresses of the plate. The influence of a change of concentrated mass on resonance frequency of the sensor plate is shown in Figure 6.

**Figure 7.**
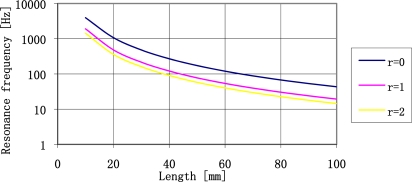
The relationship between sensor plate resonance frequency made of ST-cut quartz 0.5 mm thick, and plate length (l) and the quantity of concentrated mass (r).

It is clear that the use of concentrated mass quantities exceeding two times the mass of the plate enables decrease of resonance frequency of a plate. It is the most effective place to decrease the resonance frequency of a plate. Continuous increase of a concentrated mass does not substantially decrease the resonance frequency of sensor plate. The further analysis of sensor parameters will be limited to such range of concentrated mass quantities. The relationship between the value of resonance frequency of a plate made of ST-cut quartz and length of the plate determined by three different concentrated mass values is shown in Figure 7.

**Figure 8.**
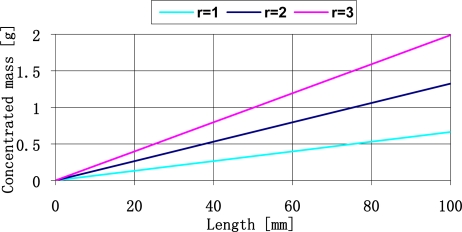
The concentrated mass quantities used in the considered sensor constructions.

From the figure presented above we may conclude that it is relatively easy to create plates of resonance frequency form 20 Hz do 4 kHz. For the 0.5 mm thick plates it is necessary to use the concentrated mass up to 1.5 g. The relation between the concentrated mass and the plate length is shown in Figure 8.

**Figure 9.**
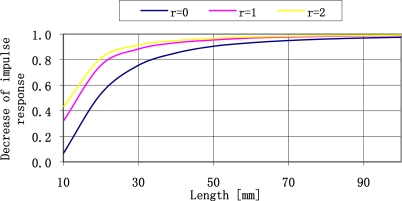
Relative decrease of impulse response amplitude after the time equal to its period in form of a function of plate length for different concentrated mass quantities (*r*).

The sensor impulse response presented by the relation Equation (25) has a damped character. Its fast fading can impose an upper limit on the resonance frequency. The damping value depends on the geometry of the plate and the equivalent damping coefficient. In order to simplify the illustration the impulse response damping measure has been introduced as a relative decrease of its quantity after one period. The relation of impulse response damping in the form of a function of length of ST-cut quartz plate for three different concentrated mass values is presented in Figure 9.

**Figure 10.**
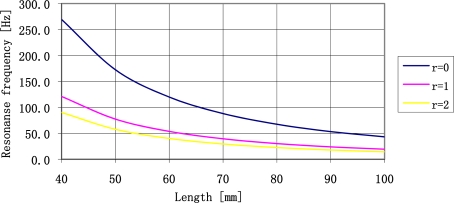
The relation between resonance frequency of a sensor plate and plate length and the value of concentrated mass.

For plates longer than 40 mm loaded with a concentrated mass equal to the mass of the plate (r = l) the damping of free vibrations of the plate is relatively slow. The impulse response of shorter plates is dampened relatively fast. This is why it seems to be beneficial to possibly use long plates loaded with a concentrated mass equal to the plate mass. The value of resonance frequencies of plates that can be manufactured has changed. It seems that the range of resonance frequency plates available to use is limited to the range from 20 Hz to 250 Hz. The parameters of resonance frequencies of the plates in the aforementioned range are shown in Figure 10.

The above-mentioned considerations may be summarized as follows: SAW vibration sensors, which are designed to be used with the impulse response, may be manufactured with 0.5 mm thick ST-cut quartz. With the use of the concentrated mass equal to the mass of a plate it is possible to reach the natural frequencies of the plates from 20 Hz to 250 Hz. In the next section a design of a SAW delay line for a sensor will be discussed.

## 3. Delay Line for SAW Vibration Sensor

**Figure 11.**
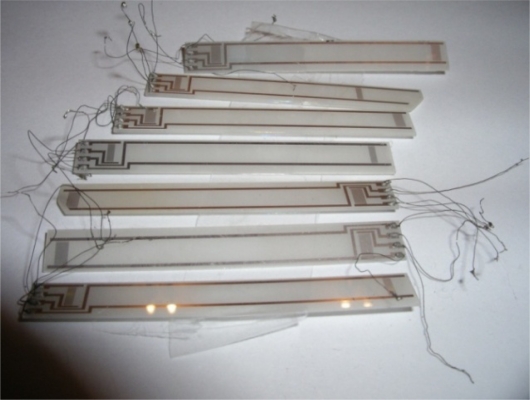
Delay lines for SAW vibration sensor.

Figure 11 presents manufactured SAW delay lines for SAW vibration sensors. They consist of two cooperating interdigital transducers on a piezoelectric plate. From an electronic point of view, the line is a four-terminal network. Because of vibration sensor plate movement, electrodes feeding electrical signals to transducers should be placed on an immovable part of the plate. This ensures proper strength of electrical contacts to the electrodes. The electrodes are long and have a specified effective resistance. Because of plate movement, its casing is different than in classic SAW filters. These structural components are characteristic for SAW delay lines applied for vibration sensors. They cause an increase in signal going directly from input into line output and increase in-line loss.

Proper design of interdigital transducers requires knowledge of piezoelectric parameters of ST-cut quartz. Table 2 presents these parameters. Main parameters of ST-cut quartz are good temperature properties and low values of electromechanical coupling and dielectric permittivity. Both of them were a decisive factor when choosing ST-cut quartz as a material for the sensor plates.

Relation between relative time delay changes of the SAW Δτ/τ and temperature *T* describes the following dependence:

(25) $$\frac{\Delta \tau }{\tau }=-34\cdot 10^{-9}{\left(T-T_{0}\right)}^{2}$$

where:

*T* —Temperature [Kelvin degree].
*T*_0_ —Return temperature (295 Kelvin degrees for ST-cut quarz).

**Figure 12.**
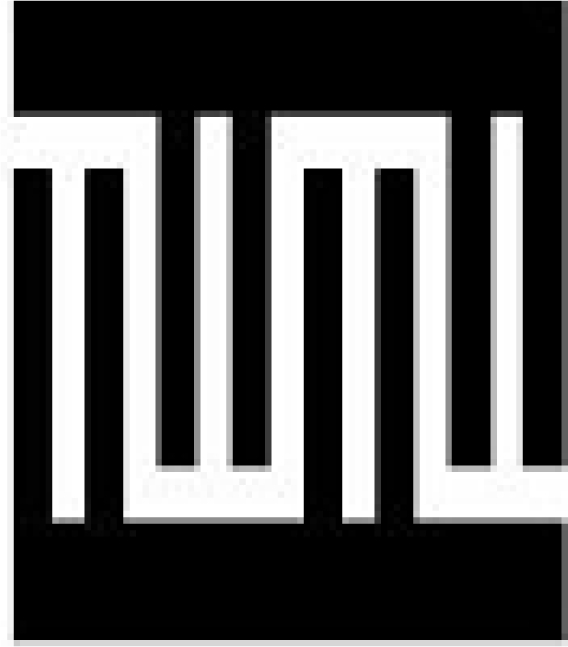
The interdigital transducer structure.

The delay line has been designed as two identical straight periodic interdigital transducers (IDTs) with double electrodes. Such a transducer structure enables to use their operation on the third harmonic. Figure 12 presents a transducer electrode unit.

**Figure 13.**
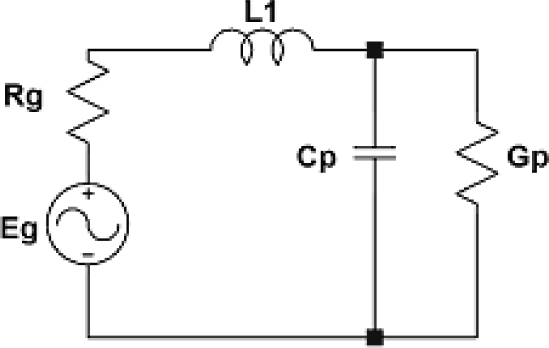
Transducer matching circuit.

The low value of electromechanical coupling for ST-cut quartz causes high losses in mismatch of its input impedance up to 50 Ω. In order to minimize the losses an operation mode of IDts has been set to match 50 Ω impedance for 74 MHz frequency. Figure 13 presents the way the transducer has been matched.

An element which matches the transducer of conductance *G_p_* and capacity *C_p_* to impedance *R_g_* = 50 Ω is inductance *L_1_*. The analysis of transducer matching in the circuit in Figure 13 leads to dependence where on transducer conductance there is emitted available power:

(26) $$\frac{G_{P}}{G_{p}^{2}+\omega^{2}C_{p}^{2}}=R_{g}=50\Omega$$

From the above dependence, using transducer electric circuit model [24] and substrate parameters (Table 2) the number of transducer electrodes and transducer aperture have been calculated. Transducer operation on the third harmonic has been used. For ST-cut quartz a medium frequency of line operation, 74 MHz, has been chosen. Electrode width and gaps between electrodes amounted to 16 μm, and length of surface wave amounted to 37 μm. For such parameters the following has been obtained: number of transducer electrodes N = 25 pairs and 2.5 mm aperture.

**Figure 14.**
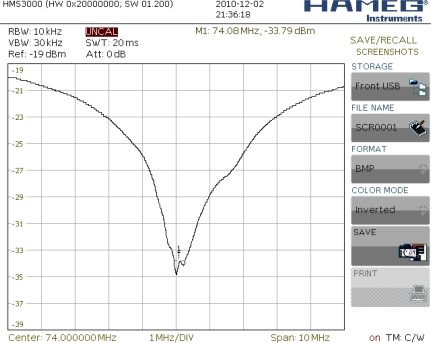
Value of coefficient of reflection from matched transducer *versus* frequency.

A transducer matching for 50 Ω impedance has been performed by measuring the coefficient of reflection. Figure 14 presents the change in value of the coefficient of reflection from the transducer in a circuit of impedance 50 *versus* frequency. For matching, an inductance of value *L*_1_ = 900 *nH* has been used.

**Figure 15.**
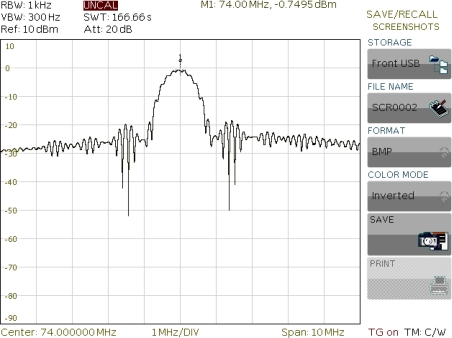
Amplitude characteristic of SAW-VS after transducers matching.

Experimental frequency characteristic amplitude of SAW vibration sensor (SAW-VS) presented in Figure 15.

**Figure 16.**
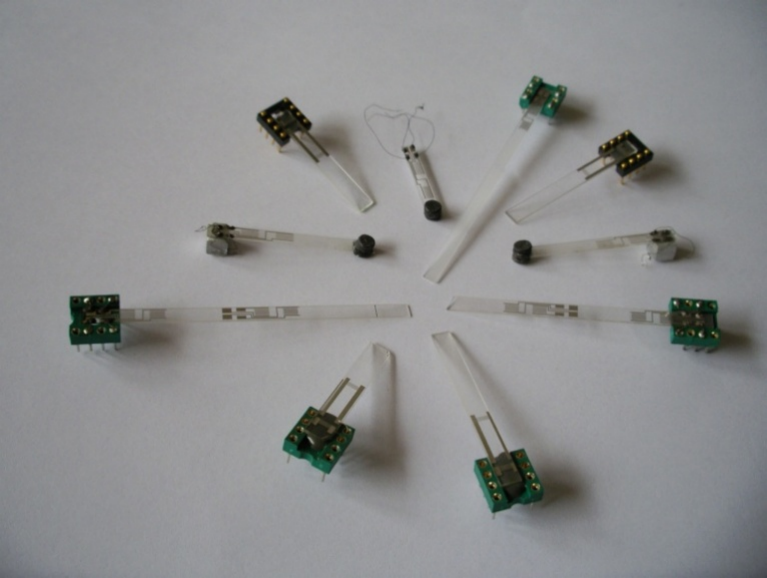
SAW delay lines ready for being assembled in vibration sensor.

Line losses after matching on frequency of 74 MHz amounted to −22 dB. SAW delay lines to be assembled in vibration sensor are presented in Figure 16.

Performed on ST-cut quartz plates and 0.5 mm thick, SAW delay lines constitute a mechanical set and (mechanical-electrical) transducer of a vibration sensor. The last part of a vibration sensor is an electric circuit which gives the possibility to measure time delay changes of a SAW. Experimental results concerning verification of the presented above theory of modeling of sensor plate are presented in the next section.

## 4. Experimental Examinations of Sensor Plates: Results

**Figure 17.**
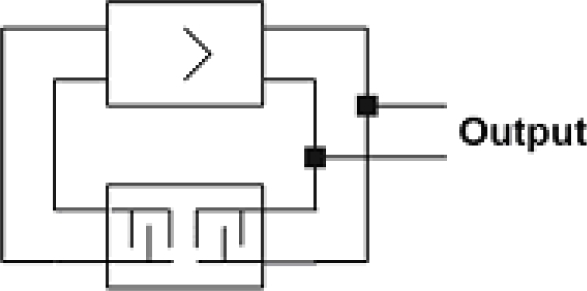
Scheme of equivalent circuit of a SAW generator with feedback loop.

Usually, time delay change in SAW sensors is measured by means of two methods [25–28], *i.e*., with a SAW generator and with a phase detector. The first one is based on the SAW generator frequency changes which have been made on the basis of the above discussed SAW delay line. The generator operates in feedback loop circuit. Figure 17 presents a scheme of the SAW generator.

**Figure 18.**
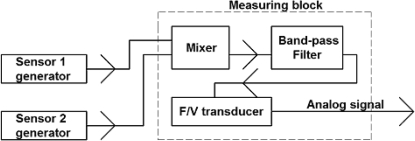
Block diagram of SAW vibration sensor.

On the basis of the presented SAW delay lines two generators have been made. In each of them different vibrating plates were applied, one with 22 Hz, the other with 100 Hz resonance frequency. Generators operated on 73.8 MHz and 73.9 MHz. A 100 kHz difference between generators enabled their operation in the unit presented in Figure 18. The output signal from the measuring block through oscilloscope was loaded into a PC using the Agilent VEE software. Its amplitude was proportional to the frequency difference of both generators.

**Figure 19.**
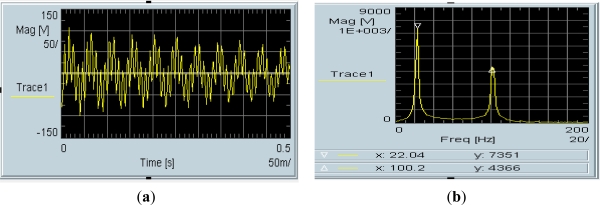
Impulse response (**a**) of a set of two cooperating SAW sensors and its spectrum (**b**).

A fragment of the impulse response and its spectrum for the set of two activated sensors are presented in Figure 19. Impulse response Equation (19a) is a sum of two fading periodic time signals. Its spectrum Equation (19b) has been obtained by the Fourier transform module of the Agilent VEE software. Resonance frequencies of the spectrum run amount to 22 Hz and 100 Hz, respectively.

Experimental results published in [14,23] have been made before the theory presented in Section 2 of mechanical unit of vibration sensor. Theoretically, the run of impulse response spectrum is a sum of frequency amplitude characteristics of both SAW sensors. Let us compare the results with theoretical amplitude of frequency response of both sensors presented in Figure 4. Runs of experimental spectra of impulse responses of sensors (Figure 19) and theoretical resonance characteristics of sensor plates (Figure 4) are characterized by high selectivity. Relative values of their amplitudes cannot be comparable because of unknown value of their vibration activation. The results confirm that from the technical point of view, plate vibrations have frequency equal only to the first harmonic.

## 5. Design of Electronic Warning System

The obtained results confirm the possibility to use SAW vibration sensor plates not only to record vibrations but also to identify each sensor in the system. Because of the high selectivity of frequency responses, band-pass filters with mean frequencies equal to the resonance frequencies of sensor plates can be applied. Such properties of sensors can be used in a system monitoring vibrations in the sensor area. Such a system should consist of several SAW sensors. It should be relatively easy to connect the sensors to the system.

**Figure 20.**
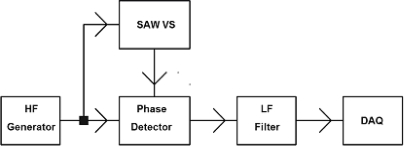
Measurement of SAW delay changes with phase detector.

In the previous section a system with two SAW generators was shown. Now an electronic warning system that uses the other method of measuring changes in SAW time delay by phase detector will be proposed. The well-known method in literature (Figure 20) is presented for one sensor. Measuring is carried out in a system consisting of high frequency generator (HFG), the SAW-VS, phase detector (PD), low frequency filter (LF) and DAQ (data acquisition) unit.

The signal from the high frequency generator is:

(27) $$u(t)=U\;\text{sin}(\Omega_{0}t)$$

where: U—amplitude, Ω_0_—frequency HFG, is divided in two paths. First path consists of SAW-VS’s and second path goes directly to phase detector input.

The output signal of low frequency filter (LF) is described by the following dependence:

(28) $$u_{d}(t)=k_{d}U_{1}U_{2}\;\text{cos}\;\Psi_{b}$$

where:

Ψ_b_—high frequency signal phase shift by SAW vibration sensor,
*U*_1_, *U*_2_—signal amplitudes on phase detector input,
*k_d_* —phase detector constant.

**Figure 21.**
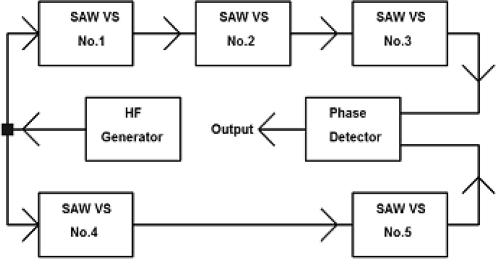
Schematic diagram of electronic warning system with five SAW-VS’s.

Changes in its value will be proportional to changes in the SAW delay caused by sensor plate movement. Because a SAW vibration sensor is an electronic four-terminal network, the unit presented in Figure 20 can be developed by cascade connected sensors as presented in Figure 21.

It consists of five SAW-VS’s placed in two paths and numbered 1–5, high frequency generator and phase detector. The first path consists of three cascades connected SAW-VS’s numbered 1–3 and second path with sensors 4 and 5. Output signals of the phase detector are:

(29) $$u_{d}(t)=k_{d}(\Psi_{1}-\Psi_{2})$$

By assuming that phase shifts in both paths originate from times delay of SAW filters and other sources of phase shift can be compensated phase shifts in both paths can be expressed as:

(30) $$\begin{array}{cc} \Psi_{1}=\sum_{n=1}^{3}\phi_{n} & \Psi_{2}=\sum_{n=4}^{5}\phi_{n} \end{array}$$

where:

(31) $$\phi_{n}=2\pi \Omega_{0}\Delta \tau_{n}$$

φ_n_—phase shift of n-th sensor,
Δτ_n_—time delay changes of n-th SAW filter.
Ω_0_—frequency HFG,

If none of the sensors is vibrating, then the difference between signal phases on phase detector input should amount to 90 degrees. The condition will be fulfilled, regardless of the amount of sensors placed in path and connection cables length and their types, by phase shifters placed in both paths. Then, using the dependence, the signal on phase detector output can be presented in the following way:

(32) $$U_{\mathrm{wy}}(t)=\sum_{n=1}^{5}B_{n}(t)\cdot e^{-\frac{\omega_{0}^{2}\tau }{2}t}\cdot \;\text{sin}(\omega_{\mathrm{rn}}t)$$

**Figure 22.**
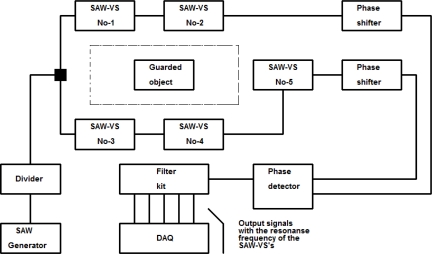
Block diagram of electronic warning system with phase shifters and electronic detection unit.

This is a sum of alternating signals of frequencies equal to the resonance frequencies of plates of all the SAW vibration sensors included in the electronic warning system. The signals’ amplitudes will depend on a type of function forcing each of the sensors to vibrate. Signal distribution is made using five-channel analog filter. Figure 22 presents the diagram of electronic warning system complemented by phase shifters and recording unit [29].

**Figure 23.**
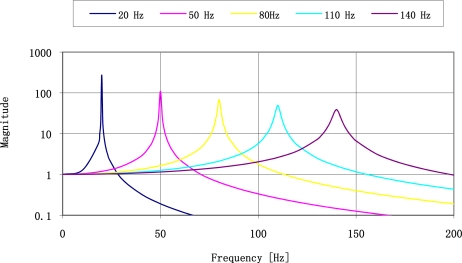
Example of frequency responses for five vibration sensors.

Figure 23 presents frequency responses of five SAW vibration sensors.

**Figure 24.**
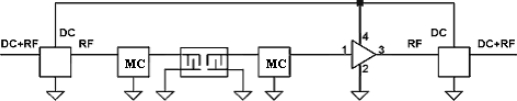
Electric operation diagram of SAW vibration detector.

Figure 24 presents an electric operation diagram of SAW vibration sensor. In order to easily connect sensor to the warning system it has been assumed that:

- test signal (74 MHz) and power supply signal 12 VDC will be sent by one coaxial cable;
- losses brought by SAW delay line will be compensated by an amplifier cooperating with the line.

The assumed solution leads to an extension of the electronic system of the SAW vibration sensor. On its input there is a test signal distribution circuit (74 MHz) and power supply signal (DC). HF signal after passing through a SAW delay line and two matching circuits (MC) is amplified to the input value level. Then, it is summed with DC power supply voltage on the sensor output.

**Figure 25.**
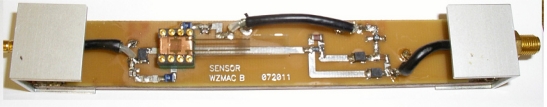
SAW vibration sensors for electronic warning system.

Tha advantage of a such a solution is that there are no changes of signal amplitude on the phase detector input if more sensors are connected to the warning system. The phase of signals in both paths of the system is changed. Its correction is made by phase shifters being integrated in the system (Figure 23). SAW vibration sensors for electronic warning system are presented in Figure 25.

**Figure 26.**
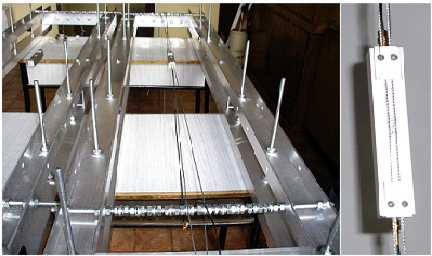
Test station for SAW vibration sensors for electronic warning system.

Signals generated by vibrating sensors are distributed on the output of a five-channel filter unit. Each of signals through the recording unit is given to the alarm central unit. It is recording vibrations of each sensor the electronic warning system consists of. To carry out experimental tests, sensors are activated on a test station shown in Figure 26.

**Figure 27.**
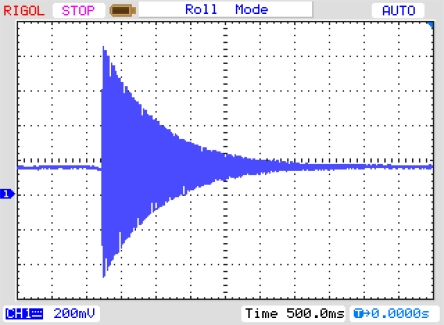
Experimental impulse response of SAW-VS.

The experimental impulse response of SAW-VS sensor has been shown in Figure 27. The plate length is *l* = 26 mm and is weighted by the concentrated mass equal to the mass of the plate (*r* = 1). Its resonant frequency was 160 Hz.

SAW vibration sensors are fixed to wire ropes. Wire ropes with adjustable tension are used to simulate vibrating strings. It makes possible to control and activate sensors according to chosen parameters, and to perform repeatability tests.

## 6. Conclusions

In this paper a feasibility study on SAW vibration sensors for electronic warning systems was presented. The SAW sensor has been based on a SAW delay line. The delay line has been made on a vibrating piezoelectric plate. A vibration model of anisotropic sensor plate has been made. By successive simplifications the description of vibrations of anisotropic viscoelastic plate has been reduced to a isotropic model with one degree of freedom. By means of the model an explicit description of plate movement has been obtained. Experimental examinations have proved that the sensor plate vibrates only with first harmonic frequency. Simple dependences have been obtained describing its value as a function of the plate geometry and volume of concentrated mass. Vibration amplitudes of higher harmonic frequencies are practically negligible. The model has been used to carry out an analysis of frequency responses of ST-cut quartz sensor plates, and an analysis of damping speed of plate impulse response. On the basis of these parameters a range of resonance frequencies of plates has been established which can be applied in the electronic detection unit. The lower range of resonance frequencies is limited by plate length. To describe it, an available ST-cut quartz crystal length of 100 mm has been assumed. The upper range of resonance frequencies is limited by the decay time of the impulse response of the sensor plate. In the analysis, the value of stress on a plate during its movement has been omitted. It must be lower than the value of dynamic critical stress presented in Table 1. The above problem has not been analyzed in the present paper.

Values of dynamic critical stress for ST-cut quartz have been determined in [22,23]. The values are not determined according to norms. They concern a series of plates cut by a wire-saw. When determining the values it turned out that the technology of plate production has a big influence on the value of dynamic critical stress. The paper [14] has proved that their value at chosen sensor structure does not limit determined frequency range of plates.

**Figure 28.**
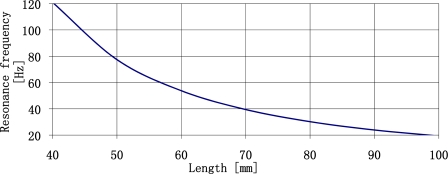
Resonance frequency of sensor plate *versus* plate length with concentrated mass equal to plate mass.

Sensor sensitivity increases along with decreasing resonance frequencies of plates. Thus, it is possible to design high sensitivity sensors. Lowering of the resonance frequency of the plate can be achieved by increasing plate length (Figure 28). It is the most effective way to lower the resonance frequency of sensor plates. For a sensor design with seismic mass equal to plate mass, a resonance frequency of 10 Hz can be obtained at a plate length of 140 mm. At a plate length of 200 mm its resonance frequency will amount to 5 Hz. The above relations allow one to design high-sensitive vibration sensors of plate resonance frequencies of several Hz.

Let us consider possible differences between a fabricated sensor and the model sensor presented in the paper. There will be two fundamental differences between them. The former concerns attaching immovable plate end. It will be viscoelastic and not rigid as in the model. The latter concerns making distributed mass instead of concentrated mass. Theoretically, the differences can be described by modifying the boundary conditions in the isotropic model of the plate. In practice, it will be hard to experimentally verify a modified model. Considering the obtained results it seems that the influence of presented differences on modeling results will be practically negligible.

The paper presents an example of applying SAW vibration sensors in an electronic warning system. Vibration sensors record vibrations in chosen areas. Signals from vibrating sensors are recorded by alarm central unit.

All vibration sensors included in the system can be placed in one area. Then the system will play a role of vibration analyzer in the area. Potentially, it is the second application of the discussed unit.

The possibility to use high-sensitive vibration sensors of resonance frequencies of several Hz is a further area of application of the sensors in monitoring vibrations of bridges and buildings whose vibration frequencies go from fractions of Hz to several Hz.

The presented application possibilities concern using SAW vibration sensor impulse responses. It is possible to apply such a sensor to measure one component of an acceleration vector. Then impulse response is a spurious signal for acceleration measurement and should be eliminated. Thus, application of sensor plate lengths with fast impulse response fade enables the design of acceleration sensors. Then, the character of plate movement is change of acceleration in time. In [30,31] designs of such type of sensors are presented. The presented model can be directly used for design purposes of such sensors. The SAW vibration sensor may be compared with two of the most often used sensors:

- piezoelectric accelerometers,
- micromechanical acceleration sensors based on silicon known as Micro Electro Mechanical Systems accelerometers.

We do not take into the consideration the impact sensors. They are light-weight and have a wide measuring band. Piezoelectric accelerometers enable acceleration measurement from parts of Hz up to some kHz. They enable measurements from 10^−3^ “g” up to 10^5^ “g” (“g” is a unit of acceleration equal to Earth’s gravity at sea level = 9.81 m/s^2^). Their sensitivity depends on their construction and has values in the 0.2 mV/“g”−0.7 V/“g” range. Their weight is not less than 3 g and not greater than 0.5 kg. The piezoelectric accelerometers natural frequencies (resonance frequencies) are high, from some kHz up to several dozen kHz. The construction of piezoelectric acceleration sensors is simple, but the measuring process is complicated. The changes of charge (on the order of pC) are measured.

MEMS accelerometers are produced in large numbers and applied in many areas: motorization, air bags, laptops, smartphones, inertial navigation, and seismic sensors. They are cheap and small. They enable acceleration measurement for constant and variable acceleration up to hundreds of Hz. They enable measurements from parts of “g” up to 10.000 “g”. MEMS accelerometers’ sensitivity depends on their construction and has the values in the range of 0.2 mV/“g”−10 V/“g”. The higher values have sensors applied in seismology. Their weight is in the of some g to 2.5 kg. The weight of sensors applied in seismology is the highest. The MEMS accelerometers’ natural frequencies (resonance frequencies) are high, up to some kHz. Their output signal is proportional to the casing acceleration, without influence of own free vibrations. The high resonance frequencies are the main reason behind the above mentioned effect. The construction of MEMS acceleration sensors is simple and the measurements are not complicated either. The changes of capacity (order about 10^−15^ F) are measured. The measuring system of MEMS acceleration sensors is similar to the measuring system of SAW vibration sensors (Figure 20). The measuring systems of MEMS acceleration sensors consist of a high frequency generator (HFG), 1 MHz, two signal paths and phase detector (PD).

The construction and measurement methods of MEMS accelerometers and SAW-VS are similar. Both have a vibrating plate as a main part. The vibrating plate of MEMS accelerometer changes the capacity of a chip capacitor and it is made of silicon. The vibrating plate of SAW-VS is made of quartz. Both plates are made of materials having similar mechanical properties. It implies similar parameters of both sensors.

The normal (resonance) frequencies SAW-VS may be in the range from 10 Hz up to hundreds of Hz. It is an important difference when we compare MEMS accelerometers and SAW-VS. Sensitivity of SAW-VS depends on parameters of plate determining resonance characteristics, length of the surface wave used in sensors and construction of sensor. We compare sensitivities for constant acceleration. The SAW-VS with the plate 65 mm × 5.5 mm × 0.5 mm loaded with the concentrated mass four times greater than the mass of the plate was tested. The measured sensitivity was 0.5 V/“g”. The sensitivity for variable acceleration may be many times greater than the above mentioned due to resonance phenomenon. Sensitivity of SAW-SV depends on length of surface wave, thickness of the plate, ratio between the concentrated mass and the mass of plate and delay time of the delay-line applied in SAW-VS. We may prepare SAW-VS with greater sensitivity. SAW-VS may also work in cascades. This enables the production of systems with higher sensitivity and smaller cross acceleration sensitivity. The compared sensors do not enable forming such systems. The construction of wireless systems with SAW-VS is possible due to very high frequency of signals used in them. The SAW-VS should thus have their place in measuring technology.

## Figures and Tables

**Table 1.** Material parameters of quartz.

**Parameter**	**ST-cut quartz**
Equivalent Young’s modulus [GPa]	76
Dynamic critical compressive stress [MPa]	80
Equivalent material damping coefficient [μs]	29.3
Density [kg/m^3^]	2,650

**Table 2.** ST-cut quartz parameters.

**Material**	**Cutting angle [degree]**	**Direction of wave propagation [degree]**	**SAW velocity V [m/s]**	**Coupling coefficient k^2^ [%]**	**Temperature coefficient**		**Relative dielectric permittivity ε**
					**a 10^−6^/K**	**b 10^−9^/K**	
Quarz ST	42.75° Y	X	3,157	0.16	0	−34	4.5
